# Improving Patient Outcomes Using Measures to Increase Discharge Rates to Home

**DOI:** 10.7759/cureus.59738

**Published:** 2024-05-06

**Authors:** Swapnil V Patel, Anne Arcidiacono, Christopher P Austin, Steven Imburgio, Joseph Heaton, Kristin DiSandro, Divya Mathur, Rocel Besa, Ellen Angelo, Brian Walch, Mohamed Bakr, Vito Buccellato, Elliot Frank, Mohammad A Hossain, Arif Asif

**Affiliations:** 1 Internal Medicine, Jersey Shore University Medical Center, Neptune City, USA

**Keywords:** healthcare administration, early ambulation, mobility technicians, discharge to home, quality improvement

## Abstract

Background

Post-acute care (PAC) centers are facilities used for recuperation, rehabilitation, and symptom management in an effort to improve the long-term outcomes of patients. PAC centers include skilled nursing facilities, inpatient rehabilitation facilities, and long-term care hospitals. In the 1990s, Medicare payment reforms significantly increased the discharge rates to PAC centers and subsequently increased the length of stay (LOS) among these patient populations. Over the last several years, there have been national initiatives and multidisciplinary approaches to improve safe discharge rates to home. Multiple studies have shown that patients who are discharged to home have decreased rates of 30-day readmissions, reduced short-term mortality, and an improvement in their activities of daily living.

Objectives

This study aimed to investigate how multidisciplinary approaches could improve a single institution's discharge rates to home. In doing so, we aim to lower hospital readmission rates, hospital length of stay, morbidity and mortality rates, and healthcare-associated costs.

Methods

A retrospective single-institution cohort study was implemented at Jersey Shore University Medical Center (JSUMC). Data from January 2015 to December 2019 served as the control period, compared to the intervention period from January 2020 to January 2024. Patients were either admitted to JSUMC teaching faculty, hospitalists, or "others," which is composed of various medical and surgical subspecialists. Interventions performed to improve home discharge rates can be categorized into the following: physician education, patient education, electronic medical record (EMR) initiatives, accountability, and daily mobility initiatives. All interventions were performed equally across the three patient populations. The primary endpoint was the proportion of patients discharged to home.

Results

There were 190,699 patients, divided into a pre-intervention group comprising 98,885 individuals and a post-intervention group comprising 91,814 patients. Within the pre-intervention group, the faculty attended to 8,495 patients, hospitalists cared for 39,145 patients, and others managed 51,245 patients. In the post-intervention period, the faculty oversaw 8,014 patients, hospitalists attended to 35,094 patients, and others were responsible for 48,706 patients. After implementing a series of multidisciplinary interventions, there was a significant increase in the proportion of patients discharged home, rising from 74.9% to 80.2% across the entire patient population. Specifically, patients under the care of the faculty experienced a more substantial improvement, with a discharge rate increasing from 73.6% to 84.4%. Similarly, the hospitalists exhibited a rise from 69.4% to 74.3%, and the others demonstrated an increase from 79.3% to 83.7%. All observed changes yielded a p-value < 0.001.

Conclusions

By deploying a multifaceted strategy that emphasized physician education, patient education, EMR initiatives, accountability measures, and daily mobility, there was a statistically significant increase in the rate of patient discharges to home. These initiatives proved to be cost-effective and led to a tangible reduction in healthcare-associated costs and patient length of stay. Further studies are required to look into the effect on hospital readmission rates and morbidity and mortality rates. The comprehensive approach showcased its potential to optimize patient outcomes.

## Introduction

The United States has experienced significant population growth, particularly among its elderly demographic, over the past few decades [1]. This trend has placed considerable strain on the healthcare system, further compounded by the challenges posed by the COVID-19 pandemic [2]. Recent data analysis indicates a notable uptick in emergency room (ER) visits and hospital admissions among individuals aged 60 and above [3]. To address these challenges, nationwide initiatives are underway to alleviate the strain on the healthcare system, with a focus on reducing patient length of stay (LOS), improving patient satisfaction, and curbing healthcare costs [4-7].

Medicare payment reforms implemented in the 1990s led to a notable surge in discharges to post-acute care (PAC) centers [8]. In recent years, efforts have been focused on improving discharge rates to home. However, more recent government initiatives have prioritized and allocated funds to home care services to encourage higher rates of home discharge [9]. Studies supporting home discharge with home health services have shown reduced 30-day readmissions, decreased short-term mortality, and enhanced activities of daily living [9]. According to the Agency for Healthcare Research and Quality, there are significant disparities in healthcare-associated costs and hospital LOSs based on patient disposition [10]. For patients requiring routine discharge home, the average LOS is only 3.6 days. This contrasts with home health services, which average 6.2 days, and PACs, which range from seven to 13.5 days, depending on the type of PAC required [10]. The longer LOS for patients in PACs contributes to higher healthcare-associated costs. Therefore, reducing the LOS leads to substantial savings for hospitals. In addition, as hospital censuses continue to rise nationally, studies have demonstrated that a significant amount of potential hospital revenue is lost when ERs become congested and patient LOSs in the ER increase [11]. These losses can be recuperated through improved LOSs and improving hospital discharge rates to home.

The overarching goal of this quality improvement project was to assess the impact of a multidisciplinary approach on discharge rate to home within a single institution. This was accomplished with daily mobilization, physician education, patient education, and electronic medical record (EMR) initiatives constituting key components of a comprehensive approach aimed at facilitating safe discharges to home [12-14].

## Materials and methods

Quality improvement process

In an effort to improve discharge-to-home rates after consistently underperforming national averages, a multidisciplinary team at Jersey Shore University Medical Center (JSUMC) was created. The team consisted of hospital administration, department heads, attending physicians, nursing management, skilled therapists, patient care associates, and the quality improvement department. A multifaceted approach was initiated and implemented over the past three years. There was an emphasis on physician education, patient education, EMR initiatives, accountability measures, and daily mobility to improve discharge rates to home.

Setting

JSUMC is an academic medical center with a level I adult and level II pediatric trauma center distinction located in Central New Jersey with 630 licensed beds. Under the Medicine and Surgical departments, there are 464 beds. The participants in this study were located across the hospital and were under the direct care of either JSUMC faculty, JSUMC hospitalists, or JSUMC others, which encompasses multiple subspecialties under the Medicine and Surgical departments.

Overview of the project

Patients older than 18 years of age admitted to JSUMC or under observation status were included in this study. This was a retrospective study with data collected over a similar period of time prior to and post-interventions implemented by a multidisciplinary team at JSUMC. This quality improvement project was exempt from the Institutional Review Board oversight as no patient identifiers or demographics were collected in this study.

Interventions

A comprehensive approach was adopted to enhance discharge rates to home, involving various interventions. Initially, a multidisciplinary team analyzed data and generated reports detailing discharge rates to home across different provider groups and individual practitioners. These reports were then shared with physician leaders, who facilitated discussions within their teams to understand the variations in discharge rates. From January 2020 to January 2024, multiple initiatives were implemented simultaneously and are found in Table 1. Data from January 2015 to December 2019 served as the control period, compared to the intervention period from January 2020 to January 2024.

**Table 1:** Implemented initiatives to enhance discharge rates to home. PACs: post-acute care facilities, BMAT: bedside mobility assessment tool

Intervention performed	Description of intervention
Physician education	Education focused on the available home care services, the advantages of daily mobilization, the benefits of home discharge versus PACs, and the implications of reduced length of stay and healthcare-associated costs.
Patient education	In an effort to improve patient comfort in being discharged home, every patient received an admission packet, which now included a brochure highlighting the benefits of home discharge and daily mobilization.
Mobility-related order sets	Enhanced visibility of mobility-related order sets for physicians led to a noticeable increase in orders for activities, such as "out of bed" and "ambulate with patients every shift," necessitating additional investments in daily mobility technicians.
Daily mobility	Mobility technicians were hired and deployed across six medical-surgical floors to assist patients with daily mobility exercises. In addition, the hospital made significant investments in acquiring more Hoyer lifts and physical therapy equipment to facilitate patients' mobility. Patients were safely selected for the mobility tech program based on their daily BMAT scores of 3 or 4. Technicians reviewed with the bedside nurse for any clinical changes that would make ambulation contraindicated.
Accountability	Regular bi-monthly reports were instituted to track discharge rates to home by providers, enabling physician leaders to identify any recurring patterns that might warrant further educational initiatives or interventions.

The cornerstone of this project was the hiring of mobility technicians to prevent deconditioning from prolonged bed rest. They were hired and employed across six medical-surgical floors after completing a standardized training course. They trained directly under the guidance of licensed physical therapists and registered nurses. The training course included lifting, mobility techniques, and safe transfer skills. Training was also provided on the safe and proper use of assistive equipment, with guidance on educating the interprofessional team and family on the importance of daily mobility.

Inclusion criteria

The inclusion criteria for patients include the following: (1) adult patients ≥18 years of age; (2) admitted to JSUMC as an inpatient, outpatient in bed, or observation on a designated medical surgical floor with mobility technicians; (3) an activity level of “up as tolerated” or “up with assistance” as ordered by the primary physician; (4) assessment of a physical therapy consult is needed prior to MT intervening as determined by the primary physician; and (5) patients requiring no more than minimal assistance required for ambulation at baseline as screened by the patient's primary nurse.

Exclusion criteria

The exclusion criteria for patients included those who left against medical advice, were readmitted within 30 days, or transferred to an outside facility.

Primary outcome

The primary outcome of this study was the proportion of patients discharged to home.

Statistics

Descriptive statistical methods were utilized to analyze the large population. The data were analyzed using a comparison of mean ± standard deviation (SD). A Z-test was utilized and an alpha (p) value was calculated. An alpha (p) value ≤0.05 was considered statistically significant. Data were summarized as median (interquartile range (IQR)) and percentages. Statistical analyses were performed using IBM SPSS Statistics for Windows, version 24.0 (released 2016, IBM Corp., Armonk, NY).

## Results

Rates of discharge to home

There were a total of 190,699 patients, divided into a pre-intervention group comprising 98,885 individuals and a post-intervention group consisting of 91,814 patients. Following the interventions, there was a noteworthy increase in the proportion of patients discharged home, rising from 73.6% (n = 98,885) to 80.2% (n = 91,814) across the entire patient population. Specifically, patients under the care of faculty experienced a more substantial improvement, with a discharge rate increasing from 73.65% (n = 6,257) to 84.4% (n = 6,764). Similarly, hospitalists exhibited a rise from 69.4% (n = 27,167) to 74.3% (n = 26,075), and others demonstrated an increase from 79.3% (n = 40,637) to 83.7% (n = 40,767). All data can be found in Table 2.

**Table 2:** Comparison of results, pre and post intervention, from the faculty, hospitalists, others, and overall The data were analyzed using a comparison of mean ± standard deviation (SD). A Z-test was utilized and an alpha (p) value was calculated. An alpha (p) value ≤ 0.05 was considered statistically significant, and all observed changes yielded a p-value < 0.001.

	Pre intervention (n = 98,885)	Post intervention (n = 91,814)	P-value	% change
Faculty discharge rates to home	73.65% (n = 6,257)	84.4% (n = 6,764)	P value < 0.001	14.60%
Hospitalists discharge rates to home	69.4% (n = 27,167)	74.3% (n = 26,075)	P value < 0.001	7.06%
Other discharge rates to home	79.3% (n = 40,637)	83.7% (n = 40,767)	P value < 0.001	5.55%
Overall hospital discharge rates to home	73.6% (n = 98,885)	80.2% (n = 91,814)	P value < 0.001	8.97%

The data was analyzed using a comparison of means. A Z-test was utilized and an alpha (p) value was calculated. An alpha (p) value ≤ 0.05 was considered statistically significant, and all observed changes yielded a p-value < 0.001.

## Discussion

This study effectively demonstrated a statistically significant improvement in discharge rates to home by employing a multidisciplinary approach involving various hospital disciplines. While many patients necessitate PAC options, such as home health, skilled nursing facilities, inpatient rehab placements, or long-term acute care facilities; research indicates that over 40% of these patients are covered by Medicare, and they tend to experience double the inpatient hospital stay compared to those discharged home [14-15]. Moreover, reducing the length of hospital stays enables healthcare systems to enhance bed turnover rates and alleviate strain on critical resources, including nursing staff [16]. Previous investigations at our institution, focusing on daily mobility to improve LOS rates, also demonstrated statistically significant reductions in patient stays [12]. These shortened stays result in substantial healthcare cost savings for both patients and healthcare systems.

Prolonged inpatient stays can heighten the risk of hospital-acquired infections, falls, venous thromboembolism, and extended periods of immobility [17]. It is crucial to note the rates of acquired infections and complications associated with PAC discharges versus home discharges. The 2021 Technical Report from the Centers for Medicare & Medicaid Services (CMS) regarding the Skilled Nursing Facility Healthcare-Associated Infections Requiring Hospitalization revealed a positive correlation between the Potentially Preventable 30-Day Post-Discharge Readmission Measure (PPR) and Skilled Nursing Facility Healthcare-Associated Infections (SNF HAI) [14-15].

While optimizing patient care to reduce inpatient hospital stays, attention must also be paid to the financial burden of PAC discharges, primarily borne by Medicare. An analysis by Tian et al. in 2013 indicated that approximately 22.3% of inpatients were discharged to PAC settings, with Medicare holding a significant financial stake of 41.7% [10]. A recent study by Werner et al., analyzing Medicare patient data from 2010 to 2016, demonstrated a statistically significant lower Medicare payment for patients discharged home compared to those sent to a skilled nursing facility [18]. With over $60 billion of Medicare funding allocated to post-discharge placement, there is a growing need for quality measures to ensure optimal patient outcomes [19].

Hospital readmission rates for PAC facilities and those discharged home are critical quality indicators affecting patient care and reimbursement, as emphasized by CMS through payment adjustment factors. Recently, CMS reported in their 2022 Skilled Nursing Facility 30-Day All-Cause Readmission Measure that almost 15,000 patients admitted to SNFs had a readmission rate of 20.1% [14-15]. This underscores the advantage of discharging patients home rather than to a facility.

Given the risks associated with longer hospital stays following PAC discharge, a safe transition to home becomes paramount to improving patient outcomes, reducing short-term mortality, and minimizing readmissions. A multidisciplinary approach, incorporating various interventions to educate patients on the importance of discharge placement throughout their inpatient stay, has shown statistically significant improvements in home discharge rates. Outside institutions can continue to build on these interventions through a multidisciplinary approach. Further research is warranted to elucidate the reduced financial costs of home discharge, analyze the evolving discharge planning landscape across US hospitals, and determine effective long-term implementation strategies. 

Limitations

There are several limitations to our study. First, the single-center study design may restrict the generalization of our results to other patient populations at different institutions. Second, specialists and surgical groups under "others" have guidelines in place and include the same stay admissions and discharges for surgical procedures, which may have skewed the data. Lastly, patients were not followed up on once discharged, and it is unclear if patients discharged home in this single institution had better long-term results than those discharged to PACs.

## Conclusions

By deploying a multifaceted strategy that emphasized physician education, patient education, EMR initiatives, accountability measures, and daily mobility, there was a statistically significant increase in the rate of patient discharges to home. These initiatives not only proved to be cost-effective but also led to tangible reduction in healthcare-associated costs, and patient LOS. Further studies at our institution are required to look into the effect on hospital readmission rates and morbidity and mortality rates. However, outside studies have already been done and have shown that home discharge with home health services has been associated with reduced 30-day readmissions, decreased short-term mortality, and enhanced activities of daily living. The comprehensive approach utilized at our institution showcased its potential to optimize patient outcomes, and further research directions should be focused on absolute cost savings and reductions in length of stay. 
